# Increased tortuosity of basilar artery might be associated with higher risk of aneurysm development

**DOI:** 10.1007/s00330-020-06917-3

**Published:** 2020-05-13

**Authors:** Kornelia M. Kliś, Roger M. Krzyżewski, Borys M. Kwinta, Bartłomiej Łasocha, Paweł Brzegowy, Krzysztof Stachura, Tadeusz J. Popiela, Radosław Borek, Jerzy Gąsowski

**Affiliations:** 1 Faculty of Medicine, Jagiellonian University Medical College, Kraków, Poland; 2 Faculty of Computer Science, Electronics and Telecommunications, AGH University of Science and Technology, Kraków, Poland; 3 TENSOR- Team of NeuroSurgery-Oriented Research, Jagiellonian University Medical College, Kraków, Poland; 4 Department of Neurosurgery and Neurotraumatology, Jagiellonian University Medical College, Macieja Jakubowskiego 2 Street, 30-688 Kraków, Poland; 5 Department of Radiology, Jagiellonian University Medical College, University Hospital, Kraków, Poland; 6 1st Department of Internal Medicine with Cardiology Subdivision, Blessed Marta Wiecka District Hospital, Bochnia, Poland; 7 Department of Internal Medicine and Gerontology, Jagiellonian University Medical College, Kraków, Poland

**Keywords:** Angiography, Basilar artery, Intracranial aneurysm

## Abstract

**Objectives:**

We analysed tortuosity of basilar artery (BA) to determine its relationship with the presence of aneurysm.

**Methods:**

We retrospectively analysed 71 patients with BA aneurysms along with 71 age- and risk factors-matched control patients without BA aneurysm. From patients’ medical records, we obtained their history including previous and current diseases and medications. For each patient, we calculated relative length (RL), sum of angle metrics (SOAM), triangular index (TI), product of angle distance (PAD) and inflexion count metrics (ICM). We used *t*-test and Mann-Whitney *U* test for continuous variables and *χ*^2^ test for dichotomised variables. To find independent predictors of BA aneurysm, we employed logistic regression analysis.

**Results:**

We found significant positive correlation between age and SOAM (*R* = 0.195, *p* = 0.02) and PAD (*R* = 0.199, *p* = 0.018). Our study also showed that patients with BA aneurysm had significantly higher SOAM (0.21 ± 0.16 vs. 0.11 ± 0.08; *p* < 0.01), PAD (0.30 ± 0.19 vs. 0.18 ± 0.11; *p* < 0.01), TI (0.23 ± 0.23 vs. 0.10 ± 0.16; *p* < 0.01) and ICM (0.20 ± 0.16 vs. 0.15 ± 0.11; *p* = 0.045). In multivariate logistic regression analysis, after adjustment for all possible confounders, SOAM (OR = 1.086; 95% CI 1.046–1.136; *p* < 0.01) and TI (OR = 1.004; 95%C: 1.002–1.006; *p* < 0.01) remained independently associated with higher risk of BA aneurysm.

**Conclusions:**

Increased tortuosity of BA is associated with higher risk of its aneurysm development.

**Key Points:**

*• Basilar artery sum of angle metrics and product of angle distance are correlated with age.*

*• Basilar artery tortuosity is independently associated with higher risk of its aneurysm development.*

*• Basilar artery tortuosity is positively correlated with its diameter and bifurcation angle.*

## Introduction

Tortuosity of blood vessels is a common angiographic finding, which can occur in every organ system [1, 2]. Increased tortuosity results from changes in mechanical factors of blood flow, such as elevated blood pressure [3], reduced axial tension and artery elongation [1]. It was also proved to be associated with systemic diseases, such as arterial hypertension [3] and diabetes mellitus [4]. Additionally, as tortuosity can be caused by vessel wall weakening, its increase can indicate the presence of vascular pathologies [5, 6].

Higher arterial tortuosity promotes haemodynamic changes of blood flow. Tortuous arteries are characterised by decreased perfusion pressure, lower wall shear stress (WSS) and prolonged relative residence time (RRT) [7]. Such changes in haemodynamics cause vessel wall impairment [8, 9], which might lead to aneurysm development. In other studies, higher tortuosity was linked to aortic aneurysm presence and risk of rupture [10], as well as to development of splenic artery aneurysm [11]. In our previous study, we have also proved that aneurysms of the middle cerebral artery (MCA) [12], anterior communicating artery (ACA) [13] and internal carotid artery (ICA) [14] are also linked to its tortuosity. However, these associations differed in terms of all used tortuosity descriptors. Therefore, we decided to similarly analyse tortuosity of the basilar artery (BA) to determine its relation with the presence and risk of aneurysm rupture.

## Materials and methods

### Patients

We retrospectively analysed data of 142 patients hospitalised between January 2014 and April 2018. Our study group consisted of 71 patients with saccular BA aneurysm and 71 control patients matched for age and risk factors, such as hypertension, diabetes mellitus and smoking. Aneurysm presence was confirmed by digital subtraction angiography (DSA). Patients’ imaging data were obtained prior to endovascular treatment. The presence of SAH was confirmed based on computed tomography scan. From patients’ medical records, we obtained their medical history, including previous and current diseases and medications. The study protocol was approved by the local bioethical committee and all patients gave informed consent. The data that support the findings of this study are available from the corresponding author upon reasonable request.

### BA tracking and tortuosity descriptors

A series of image transformations was performed to detect BA course on the anterior-posterior projection of each patient’s DSA. First, we subtracted bone structures and performed gamma correction to increase visibility of blood vessels. Then, multiscale vessel enhancement filter was applied to find all vessel-like structures on the image. We also binarised the images and finally applied canny edge detection to detect vessel edges. Then, we used the method described by Yin et al [15] to extract a curve representing the BA course. In the next step, for each patient’s BA course, we calculated five tortuosity descriptors. The first of them is relative length (RL), defined as:

$$ \mathrm{RL}=\frac{l}{l_c} $$

where *l*_*c*_ is the curve length and *l* is the length of the straight line between the start and end points of the curve. Next is sum of angle metrics (SOAM), for which we divide a curve into subcurves of equal length. Then, for each subcurve, we calculate supplementary of the angle between lines connecting its centre and ends. SOAM is defined as:

$$ \mathrm{SOAM}=\frac{\ {\sum}_{i=1}^n\left(180{}^{\circ}-{\varphi}_i\right)}{l_c} $$

where *휑* indicates measured angles and *n* is the count of these angles. The third descriptor is the product of angle distance (PAD), defined as:

$$ \mathrm{PAD}=\frac{\mathrm{SOAM}}{\mathrm{RL}}. $$

**Fig. 1:**
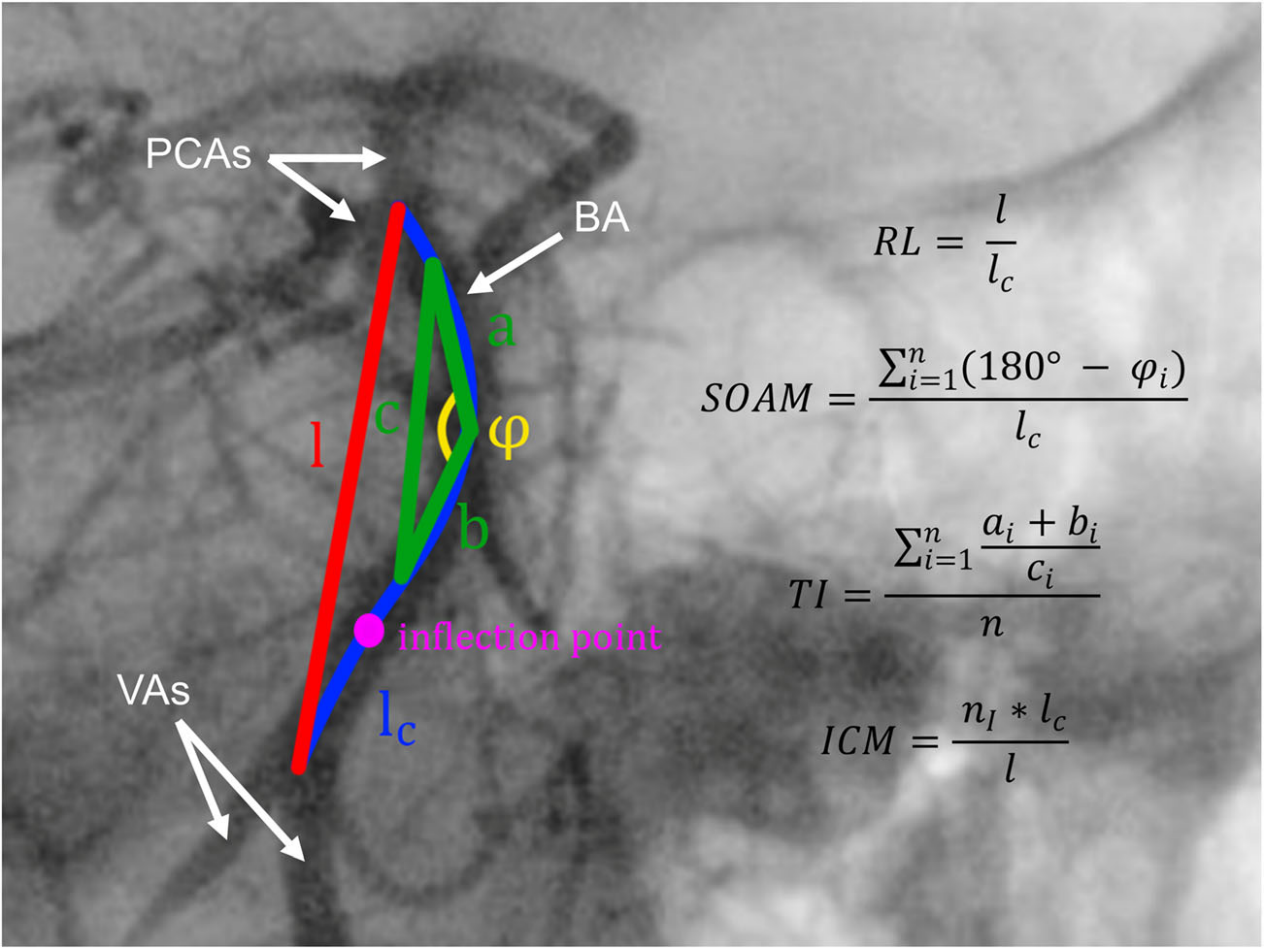
Illustration of basilar artery (BA) tracking and tortuosity descriptors calculation. RL, relative length; SOAM, sum of angle metrics; PAD, product of angle distance; TI, triangular index; ICM, inflexion count metrics; *n*, number of angles on BA course; *n*_I_, number of inflexion points on BA course; PCAs, posterior cerebral arteries; Vas, vertebral arteries. Marked angle, triangle and inflexion point are exemplary and are applicable to all parts of the artery. *l*, length of straight line between starting and ending point of analysed artery segment; *l*_c_, absolute length of analysed artery segment; a, b and c, sides of triangle constructed on angle of analysed artery segment; α, angle on analysed artery segment

The fourth is triangular index (TI), for which the curve is again divided into equal subcurves. Then, a triangle is built with vertices on each end of the subcurve and in its middle point. TI is defined as:

$$ \mathrm{TI}=\frac{\ {\sum}_{i=1}^n\frac{a_i+{b}_i}{c_i}}{n} $$

where *n* is the number of subcurves, *a* and *b* are sides of triangles and *c* is its base. The last of the tortuosity descriptors is the inflexion count metrics (ICM), defined as:

$$ \mathrm{ICM}=\frac{\ {n}_I\times {l}_c}{l} $$

where *n*_*I*_ represents the number of the curve’s inflexion points. All used tortuosity descriptors are all presented in Fig. 1.

### Additional measurements

We additionally measured diameters of both P1 segments of posterior cerebral arteries (PCAs) and diameters of both vertebral arteries (VAs) at a distance of 5 mm from the origin of BA. We also measured the diameter of BA in three locations: 2 mm from its origin, 2 mm before its bifurcation and halfway between those two locations. Additionally, we obtained the angle of BA bifurcation and its configuration, introduced by Rashad et al [16] including hands-up, hands-neutral and hands-down configurations, as well as its mixed variants (hands-up/hands-neutral, hands-up/hands-down, hands-neutral/hands-down). Among patients with BA aneurysm, we measured aneurysm dome height and width, as well as its neck size. We also calculated dome-to-neck ratio, defined as aneurysm dome height/neck size.

### Statistical analysis

The database management and statistical analysis were performed with RStudio version 8.5 for Windows (RStudio, Inc).

We used the Shapiro-Wilk test to assess normality. For comparisons of continuous variables, we used the *t*-test for normally distributed variables and Mann-Whitney *U* test for non-normally distributed variables. We used the *χ*^2^ test for dichotomised variables. To assess correlation between continuous variables, we used Pearson’s or Spearman’s correlation test, for normally and non-normally distributed variables, respectively. We express continuous variables as mean ± standard deviation. To find factors independently associated with the presence of the BA aneurysm, we employed logistic regression analysis, with and without adjustment for possible confounders. All significance tests are two tailed and the *p* value of < 0.05 has been considered significant.

## Results

### Study group characteristics

Our study group consisted of 142 patients and 90 (63.38%) of them were females. Th mean age of the study group was 58.45 ± 12.38 years. In terms of artery diameters, the mean left VA diameter was 2.95 ± 0.88 mm, mean right VA diameter was 2.67 ± 3.48 mm, mean diameter of BA was 3.26 ± 2.09 mm, mean diameter of right PCA was 1.66 ± 0.64 mm and mean diameter of right PCA was 1.76 ± 0.62 mm. Mean BA bifurcation angle was 131.61° ± 39.60°. The most common configuration of BA bifurcation was hands-up (39.44%), then hands-up/hands-neutral (25.35%), hands-up/hands-down (11.97%), hands-neutral (9.86%), hands-neutral/hands-down (7.75%) and hands-down (5.63%). Among patients with BA aneurysm, mean aneurysm dome size was 7.83 ± 4.45 mm and mean neck size was 3.42 ± 2.62 mm. A total of 33 (46.48%) patients had ruptured aneurysm. In terms of tortuosity descriptors, mean RL of study group was 0.707 ± 0.203, mean SOAM was 0.163 ± 0.136, mean PAD was 0.240 ± 0.167, mean TI was 0.167 ± 0.208 and mean ICM was 0.176 ± 0.140.

### Association of risk factors and aneurysm presence with tortuosity

**Fig. 2:**
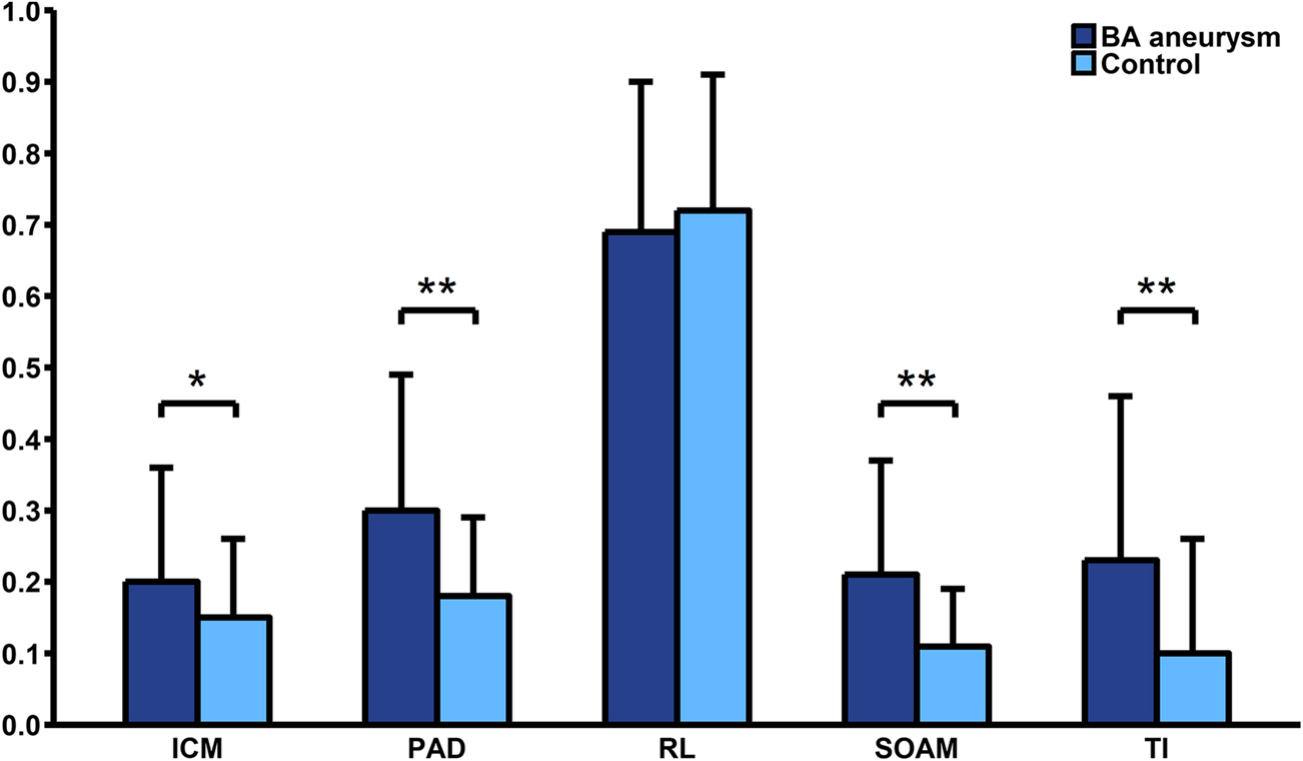
Scatter plot relating basilar artery tortuosity against age. SOAM, sum of angle metrics

**Fig. 3:**
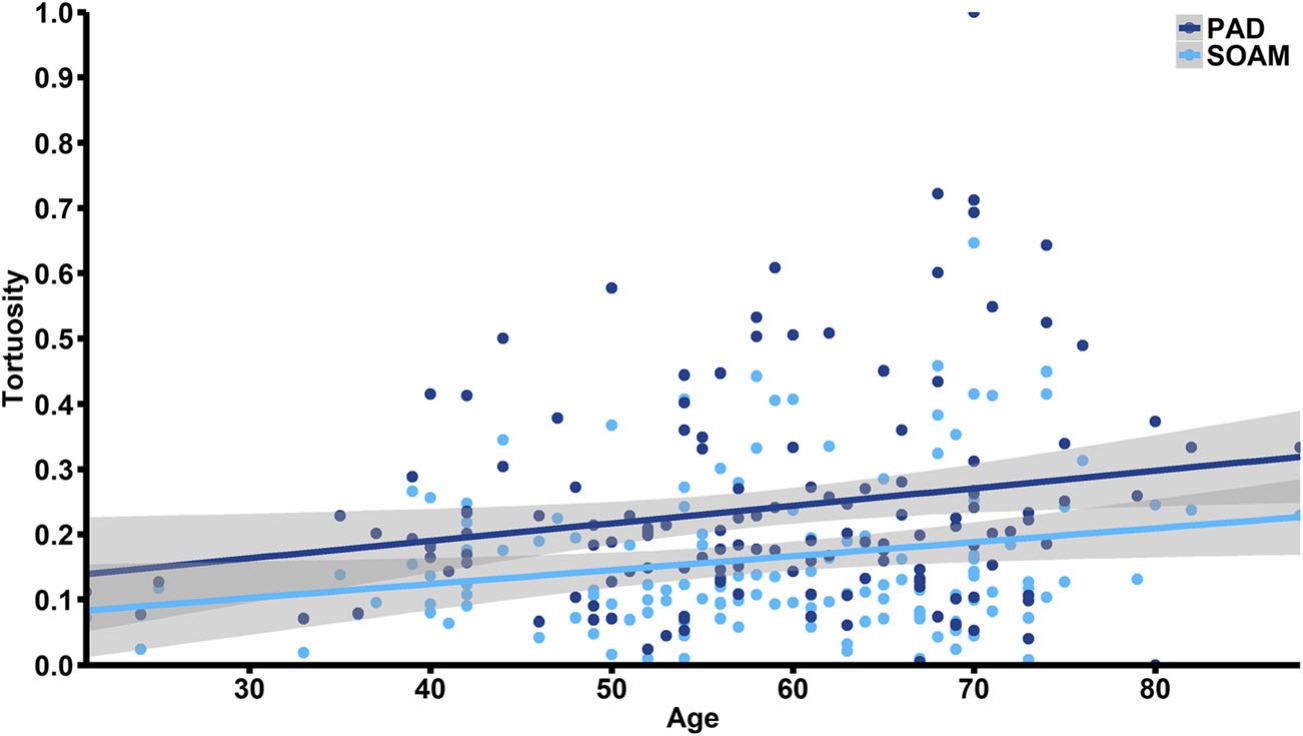
Comparison of tortuosity descriptors between patients with BA aneurysms and study group. RL, relative length; SOAM, sum of angle metrics; TI, triangular index; PAD, product of angle distance; ICM, inflexion count metrics. **p value < 0.01, *p value < 0.05

We found no significant association between aneurysm risk factors such as hypertension, diabetes mellitus, smoking or hypercholesterolaemia and tortuosity descriptors. However, we found a significant positive correlation between age and SOAM (*R* = 0.195, *p* = 0.02) and PAD (*R* = 0.199, *p* = 0.018) (Fig. 2). Our study also showed that patients with BA aneurysm had significantly higher SOAM (0.21 ± 0.16 vs. 0.11 ± 0.08; *p* < 0.01), PAD (0.30 ± 0.19 vs. 0.18 ± 0.11; *p* < 0.01), TI (0.23 ± 0.23 vs. 0.10 ± 0.16; *p* < 0.01) and ICM (0.20 ± 0.16 vs. 0.15 ± 0.11; *p* = 0.045). We found no association between RL and BA aneurysm presence (0.69 ± 0.21 vs. 0.72 ± 0.19; *p* = 0.46) (Table 1, Fig. 3). We also found no significant differences in tortuosity descriptors between patients with ruptured and unruptured aneurysm. In multivariate logistic regression analysis, after adjustment for all possible confounders, SOAM (OR = 1.086; 95% CI 1.046–1.136; *p* < 0.01) and TI (OR = 1.004; 95% CI 1.002–1.006; *p* < 0.01) remained independently associated with higher risk of BA aneurysm.

**Table 1:** Comparison of aneurysm development risk factors between study and control group. *BA*, basilar artery; *SD*, standard deviation; *PCA*, posterior cerebral artery; *VA*, vertebral artery

	BA aneurysm (*n* = 71)	No BA aneurysm (*n* = 71)	*p* value
Female gender (%)	61.97 (44)	64.79 (46)	0.73
Age (years) ± SD	58.15 ± 13.3	58.76 ± 11.47	0.77
Risk factors			
Diabetes mellitus (%)	8.45 (6)	8.45 (6)	0.58
Smoking (%)	23.94 (17)	21.13 (15)	0.73
Hypertension (%)	43.66 (31)	42.25 (30)	0.98
Ischaemic heart disease (%)	4.23 (3)	4.23 (3)	0.99
History of heart attack (%)	2.82 (2)	1.41 (1)	0.56
History of ischaemic stroke (%)	0 (0)	9.86 (7)	< 0.01
Atrial fibrillation (%)	2.86 (2)	0 (0)	0.17
Lung diseases (%)	4.23 (3)	2.82 (2)	0.65
Hypothyroidism (%)	5.63 (4)	1.41 (1)	0.17
Hypercholesterolaemia (%)	1.41 (1)	14.08 (10)	< 0.01
Current medications			
Acetylsalicylic acid (%)	7.04 (5)	16.9 (12)	0.07
Β-blockers (%)	15.49 (11)	9.86 (7)	0.31
Angiotensin-converting enzyme inhibitors (%)	15.49 (11)	11.27 (8)	0.46
AT_2_-blockers (%)	1.41 (1)	5.63 (4)	0.17
Calcium channel blockers (%)	1.41 (1)	4.23 (3)	0.31
Diuretics (%)	7.04 (5)	4.23 (3)	0.47
Steroids (%)	1.41 (1)	0 (0)	0.32
Antidiabetic therapy (%)	2.82 (2)	4.23 (3)	0.65
Insulin (%)	1.41 (1)	1.41 (1)	0.99
Heparin (%)	1.41 (1)	0 (0)	0.31
Anticoagulants (%)	1.41 (1)	2.82 (2)	0.56
Nitrates (%)	1.43 (1)	0 (0)	0.34
Statins (%)	5.63 (4)	8.45 (6)	0.51
Artery sizes			
Left PCA diameter (mm) ± SD	1.86 ± 0.71	1.66 ± 0.5	0.06
Right PCA diameter (mm) ± SD	1.69 ± 0.67	1.62 ± 0.62	0.49
Mean BA diameter (mm) ± SD	3.34 ± 1.95	3.18 ± 2.23	0.64
Left VA diameter (mm) ± SD	2.99 ± 0.87	2.91 ± 0.89	0.56
Right VA diameter (mm) ± SD	2.95 ± 4.56	2.31 ± 0.95	0.41
PCA angle (°) ± SD	135.63 ± 34.11	127.76 ± 44.12	0.24
Tortuosity descriptors			
Relative length ± SD	0.69 ± 0.21	0.72 ± 0.19	0.46
Sum of angle metrics ± SD	0.21 ± 0.16	0.11 ± 0.08	< 0.01
Product of angle distance ± SD	0.30 ± 0.19	0.18 ± 0.11	< 0.01
Triangular index ± SD	0.23 ± 0.23	0.10 ± 0.16	< 0.01
Inflexion count metric ± SD	0.20 ± 0.16	0.15 ± 0.11	0.045

### Association of artery dimensions with tortuosity

Our study showed a significant positive correlation between right PCA diameter and SOAM (*R* = 0.235; *p* = 0.043) and TI (*R* = 0.265; *p* = 0.022). Similarly, the left VA diameter was negatively correlated with RL (*R* = − 0.339; *p* < 0.01) and positively with TI (*R* = 0.415; *p* < 0.01). We also found that mean BA diameter was positively correlated with SOAM (*R* = 0.326; *p* < 0.01), PAD (*R* = 0.315; *p* < 0.01), TI (*R* = 0.280; *p* < 0.01) and ICM (*R* = 0.310; *p* < 0.01), and BA bifurcation angle was positively correlated with SOAM (*R* = 0.353; *p* < 0.01) and PAD (*R* = 0.366; *p* < 0.01) (Table 2). In terms of aneurysm size, we were unable to show a significant correlation between its dome and any of the tortuosity descriptors; however, neck size was positively correlated with ICM (*R* = 0.256; *p* = 0.03) (Table 3).

**Table 2:** Correlation between arteries measurements and basilar artery tortuosity. *RPCA*, right posterior cerebral artery; *LPCA*, left posterior cerebral artery; *BA*, basilar artery; *RVA*, right vertebral artery; *LVA*, left vertebral artery; *RL*, relative length; *SOAM*, sum of angle metrics; *PAD*, product of angle distance; *TI*, triangular index; *ICM*, inflexion count metrics

	RPCA	LPCA	Mean BA	RVA	LVA	PCA angle
RL	− 0.089	0.100	− 0.013	− 0.027	− 0.339	0.128
*p* value	0.45	0.39	0.92	0.82	< 0.01	0.27
SOAM	0.235	0.135	0.326	− 0.015	0.194	0.353
*p* value	0.043	0.25	< 0.01	0.90	0.09	< 0.01
PAD	0.214	0.149	0.315	− 0.027	0.117	0.366
*p* value	0.07	0.20	< 0.01	0.82	0.32	< 0.01
TI	0.265	0.086	0.280	0.031	0.415	0.124
*p* value	0.022	0.46	0.015	0.79	< 0.01	0.29
ICM	0.102	0.098	0.305	0.059	0.182	0.165
*p* value	0.39	0.40	< 0.01	0.62	0.12	0.16

**Table 3:** Correlation between aneurysm measurements and basilar artery tortuosity

	Dome size	Neck size	Dome/neck ratio
Relative length	− 0.020	− 0.0698	0.094
*p* value	0.87	0.57	0.44
Sum of angle metrics	0.077	0.040	0.015
*p* value	0.53	0.74	0.90
Product of angle distance	0.102	0.025	0.011
*p* value	0.40	0.84	0.93
Triangular index	0.175	0.130	0.163
*p* value	0.15	0.28	0.16
Inflexion count metrics	0.134	0.247	0.005
*p* value	0.27	0.039	0.97

## Discussion

In our study, we found a significant association between age and BA tortuosity. A similar association was found in studies concerning coronary arteries [17] and retinal vessels [18]. In terms of cerebral arteries, increase of tortuosity with age was shown for the carotid artery [19] and white matter arterioles [20]. Such correlation is most likely associated to degenerative changes in arterial walls [4, 21], as well as to higher probability of cardiovascular comorbidities.

Another finding of our study was the significant association of SOAM, TI, PAD and ICM with the presence of BA aneurysm. Kim et al in their study [22] also found such correlation. It was shown before for aortic [10] and splenic artery [11] aneurysms. In terms of other cerebral vessels, a similar association was found for ICA [14, 23], MCA [12], ACoA [13] and VA [24]. Additionally, increased tortuosity was associated with lower risk of intracranial aneurysm rupture [25] and inversely correlated with its size [13]. The association of higher tortuosity with aneurysm development most likely results from weakening of the arterial wall. The study of Rikhtegar et al showed that higher tortuosity of coronary arteries results in lower WSS and prolonged RRT [7]. Both of these changes could promote endothelial proinflammatory response and therefore lead to atherosclerotic changes in arterial walls [8, 26]. The weakening of arterial wall resulting from atherosclerotic plaques might further contribute to aneurysm development [8, 9]. The association of lower WSS and aneurysm formation was proved before by other authors [27]. The fact that cerebral atherosclerosis was associated with MCA tortuosity also proves such theory [28]. Lower WSS can additionally contribute to matrix metalloproteinases activation [29], which results in remodelling of the arterial wall [30]. Also, increased tortuosity might be caused by elevated blood pressure [3] and blood flow [31], which are considered risk factors for aneurysm development.

An interesting finding of this and our previous studies, in which we analysed tortuosity of MCA, ACA and ICA [12–14, 25], are differences in contribution of certain tortuosity descriptors to risk of aneurysm development. For all four arteries, we found that increased values of TI were associated with higher risk of aneurysm presence. We also found that TI was inversely correlated with aneurysm dome size [25]. In terms of SOAM and PAD, these factors were significantly lower among patients with MCA aneurysms, but significantly higher for other cerebral aneurysmal arteries. Also, ICM was significantly higher and RL significantly lower for patients with MCA and ICA aneurysms. On the other hand for patients with anterior communicating artery aneurysms, RL of ACA was significantly higher. The fact that we were unable to find a significant association between RL and BA aneurysm presence suggests that angles and inflexion points have more influence on aneurysm development risk than just deviation from straight line. Opposite to MCA, ACA and ICA course, natural BA course is straight; therefore, substantial lowering of RL can be caused by BA shaped like a single arch, which influences haemodynamics less than multiple angles. Also, as written in the study concerning ACA [14], RL might not be a suitable tortuosity descriptor for the intracranial arteries. In terms of opposite findings of SOAM influence on MCA and BA aneurysm presence, it is known that both lower and higher WSS might contribute to arterial wall disfunction leading to aneurysm formation [27]. Another explanation of differences between this study and studies concerning other cerebral arteries is the fact that posterior cerebral circulation is characterised by lower blood flow [32] than anterior circulation and worse sympathetic innervation [33], which ensures cerebral autoregulation. Therefore, due to different haemodynamics characteristic BA and other intracranial arteries might differ in susceptibility to WSS changes and arterial wall damage. Also, the study of Qiao et al showed the higher remodelling rate of atherosclerotic plaques in posterior circulation [34], which could further indicate the more significant role of atherosclerosis on aneurysm formation in that location. Additionally, the fact that influence of certain tortuosity descriptors on aneurysm presence is most similar between ICA and BA might suggest the role of artery diameter in that association.

We also found a significant positive correlation between VA, PCA and BA dimensions and BA tortuosity. A similar correlation was also found in studies concerning coronary arteries [35]. An explanation for such a finding could be the fact that both artery diameters [35] and BA bifurcation angle [36] increase with age, which is also related to tortuosity. Larger artery diameters could also indicate its walls weakening, as well as larger blood flow, which also promotes tortuosity [4, 31].

Another finding of our study was the positive association between aneurysm neck size and tortuosity descriptors. Due to our knowledge, none of the previous researchers found such a correlation. A wider aneurysm neck among patients with tortuous BA could be another indication of substantial arterial wall weakening due to the above-mentioned mechanisms. Also, the study of Qiu et al showed that aneurysm neck size is significantly associated with lower WSS [37], which further explains that correlation.

Our study has some limitations. First, our control group included patients with aneurysm in a different location other than BA, due to the fact that patients without intracranial aneurysm rarely undergo DSA. Another limitation was our inability to measure BA tortuosity before aneurysm formation; therefore, it remains unclear how aneurysm presence affects artery remodelling. Also, as BA is a rare location of aneurysm, our study group included only 71 patients with such aneurysms. Despite those limitations, we were able to precisely and objectively measure tortuosity of BA and show its association with risk of aneurysm formation.

## Conclusions

Our study showed that increased tortuosity of BA given by SOAM, PAD, TI and ICM is associated with higher risk of its aneurysm formation as well as that SOAM and TI are independently associated with BA aneurysm presence. We also showed that BA tortuosity is significantly correlated to age and VA, BA and PCA diameters. It was also associated with aneurysm neck size.

## Data Availability

The data that support the findings of this study are available from the corresponding author upon reasonable request.

## Abbreviations

ACA: Anterior cerebral artery
BA: Basilar artery
DSA: Digital subtraction angiography
ICA: Internal carotid artery
ICM: Inflexion count metric
MCA: Middle cerebral artery
PAD: Product of angle distance
PCA: Posterior cerebral artery
RL: Relative length
RRT: Relative residence time
SOAM: Sum of angle metrics
TI: Triangular index
VA: Vertebral artery
WSS: Wall shear stress
